# Mapping and ablation of left atrial roof-dependent tachycardias using an ultra-high resolution mapping system

**DOI:** 10.1186/s12872-022-02505-z

**Published:** 2022-02-16

**Authors:** Shinsuke Miyazaki, Kanae Hasegawa, Kazuya Yamao, Eri Ishikawa, Moe Mukai, Daisetsu Aoyama, Minoru Nodera, Junya Yamaguchi, Yuichiro Shiomi, Naoto Tama, Hiroyuki Ikeda, Yoshitomo Fukuoka, Kentaro Ishida, Hiroyasu Uzui, Yoshito Iesaka, Hiroshi Tada

**Affiliations:** 1 Department of Cardiovascular Medicine, Faculty of Medical Sciences, University of Fukui, 23-3 Shimo-aiduki, Matsuoka, Eiheiji-cho, Yoshida-gun, Fukui 910-1193 Japan; 2 Cardiovascular Center, Tsuchiura Kyodo Hospital, Tsuchiura, Ibaraki Japan

**Keywords:** Atrial tachycardia, Roof line, Ultra-high resolution mapping, Catheter ablation

## Abstract

**Background:**

Left atrial roof-dependent tachycardias (LARTs) are common macroreentrant atrial tachycardias (ATs). We sought to characterize clinical LARTs using an ultra-high resolution mapping system.

**Methods:**

This study included 22 consecutive LARTs in 21 patients who underwent AT mapping/ablation using Rhythmia systems.

**Results:**

Three, 13, 4, and 2 LART patients were cardiac intervention naïve (Group-A), post-roof line ablation (Group-B), post-atrial fibrillation ablation without linear ablation (Group-C), and post-cardiac surgery (Group-D), respectively. The mean AT cycle length was 244 ± 43 ms. Coronary sinus activation was proximal-to-distal or distal-to-proximal in 16 (72.7%) ATs. The activation map revealed 13 (59.1%) clockwise and 9 (40.9%) counter-clockwise LARTs. A 12-lead synchronous isoelectric interval was observed in 10/19 (52.6%) LARTs. The slow conduction area was identified on the LA roof, anterior/septal wall, and posterior wall in 18, 6, and 2 ATs, respectively. Twenty concomitant ATs among 13 procedures were also eliminated, and peri-mitral AT coexisted in 7 of 9 non-group-B patients. In group-B, the conduction gap was predominantly located on the mid-roof. Sustained LARTs were terminated by a single application and linear ablation in 6 (27.3%) and 9 (40.9%), while converting to other ATs in 7 (31.8%) LARTs. Complete linear block was created without any complications in all, however, ablation at the mid-posterior wall was required to achieve block in 4 (18.2%) procedures. During 14.0 (6.5–28.5) months of follow-up, 17 (81.0%) and 19 (90.5%) patients were free from any atrial tachyarrhythmias after single and last procedures.

**Conclusions:**

The LART mechanisms were distinct in individual patients, and elimination of all concomitant ATs was required for the management.

## Background

Atrial tachycardias (ATs) are challenging arrhythmias to control with medical treatment, and catheter ablation is a reasonable treatment option for their management [1]. A profound understanding of the mechanisms of ATs in individual patient is essential for a successful ablation, however the resolution of conventional 3-D mapping system was limited to clarify the exact mechanisms [2, 3]. The macroreentrant ATs, including left atrial (LA) roof-dependent tachycardias, are the most common ATs and creating durable linear conduction block to interrupt the reentrant circuit is required. However, few previous papers have focused on the detailed mechanisms of roof-dependent AT. Recently, ultra-high resolution maps can be created with Rhythmia mapping system (Boston Scientific, Natick, MA, USA) and a 64-electrode minibasket mapping catheter (Orion, Boston Scientific) to clarify the mechanisms of tachycardia circuit [4–14]. We aimed to characterize LA roof-dependent ATs using this system.

## Methods

### Study population

Twenty-two consecutive LA roof-dependent ATs identified in 21 patients between November 2016 and June 2020 were included. All were mapped with the Rhythmia mapping system. Non-clinical ATs (not identified prior to the procedure or induced by programmed stimulation) were not included. The study protocol was approved by the Fukui University institutional review board (No. 20180040). The study complied with the Declaration of Helsinki.

### Index procedure

In patients who had undergone previous procedures, all procedures were performed with an irrigated-tip catheter (ThermoCool SmartTouch or ThemoCool SmartTouch Surround Flow, Biosense Webster, Diamond Bar, CA, USA) under the guidance of a 3-D mapping system (CARTO3, Biosense Webster). The maximum power output was 35 W and temperature limit 43 °C.

### Mapping and ablation of ATs

The procedures were performed under uninterrupted anticoagulation therapy, and the target activated clotting time was > 300 s during the procedure. Mapping and ablation were performed without sedation or with minimal sedation. A single transseptal puncture was performed using a radiofrequency needle to map the LA [14].

The detailed electroanatomic maps of the ATs were created using the basket catheter (0.84 mm^2^ microelectrodes and 2.5-mm interelectrode distance center to center) and Rhythmia system with the same acceptance criteria and setting as our previous study [14]. Entrainment mapping was used to identify the active reentrant circuit if the cycle length was ≥ 200 ms. The LVAs area (< 0.5 mV), dense scar area (< 0.03 mV), and the conduction velocity were measured offline as reported previously [14]. The “*Lumipoint”* algorithm was used to analyze the activation maps to identify the critical isthmus of the ATs [14].

We targeted a narrow critical isthmus based on the propagation map if it was present or placed linear lesion at the LA roof area connecting the upper PVs if narrow isthmus was not present [14]. A 4-mm irrigated-tip catheter (FlexAbility St. Jude Medical, Minneapolis) (without contact force sensing) with a power of 30–35 W was used. The bidirectional conduction block was assessed by activation maps, presence of double potentials on the line, and differential pacing technique. The non-inducibility of any stable ATs by burst atrial pacing (up to 200 ms) from multiple sites without an isoproterenol infusion was the additional endpoint.

### Follow-up

The patients were monitored with continuous monitoring for 3–7 days post-procedure. The outpatient clinic visits were 1, 3, 6, 9, 12 months post-procedure, and subsequent visits consisted of a clinical interview, ECGs, and/or 24 h Holter monitoring every 3 months at our cardiology clinic. Recurrence was defined as any atrial tachyarrhythmias lasting > 30 s and the blanking period was 3 months.

### Statistical analysis

Continuous data are expressed as the mean ± standard deviation for normally distributed variables or as the median (25th, 75th percentiles) for non-normally distributed variables, and were compared using a Student’s t-test (one-way analysis of variance) or Mann–Whitney U-test (Kruskal–Wallis one-way analysis of variance), respectively. Categorical variables were compared using the chi-square test. A probability value of *p* < 0.05 indicated statistical significance.

## Results

### Patient characteristics

The median age was 73.0 (58.5–80.5) years, and 12 (57.1%) patients were men (Table 1). Five (23.8%) had structural heart disease including 2 (9.5%) patients with a history of a previous cardiac surgery for valvular heart disease. Four (19.0%) patients had sick sinus syndrome and 1 (4.8%) had atrioventricular conduction disturbances. All except for 1 patient (95.2%) had a history of AF (paroxysmal in 7 and persistent in 13 patients). During the procedure, small doses of antiarrhythmic drug therapy were continued in 7 (31.8%) procedures (Table 1).

**Table 1:** Patient characteristics

Patient, n	21
LA roof-dependent atrial tachycardia, n	22
Age, years	73.0 (58.5–80.5)
Male gender, n (%)	12 (57.1%)
Concomitant AF, n (%)	20 (95.2%)
Structural heart disease, n (%)	5 (23.8%)
Hypertrophic cardiomyopathy, n	1
Dilated cardiomyopathy, n	1
Cardiac sarcoidosis, n	1
Cardiac surgery, n (%)	2 (9.5%)
Mitral valve repair, n	1
Aortic and mitral valve repair, n	1
Sick sinus syndrome, n (%)	4 (19.0%)
AV conduction disturbance, n (%)	1 (4.8%)
Left atrial diameter, mm	42.6 ± 6.4
Left ventricular ejection fraction, %	58.8 ± 14.4
Brain natriuretic peptide, pg/ml	89.1 [48.0–157.0]
Antiarrhythmic drugs before procedure, n (%)	7 (31.8%)
Flecainide 100 mg/day, n	1
Propafenone 300 mg/day, n	1
Bepridil 100 mg/day, n	2
Amiodarone 100 mg/day, n	3

*AF* atrial fibrillation, *AV* atrioventricular, *LA* left atrial

The procedures were the index, second, third, and fourth ablation procedures in 5 (22.7%), 8 (36.4%), 8 (36.4%), and 1 (4.5%) ATs, respectively (Table 2). Among the 22 ATs, 3 were in cardiac intervention naïve patients (Group-A), 13 after a previous LA roof line ablation (Group-B), 4 after AF ablation without LA linear ablation (Group-C), and 2 after cardiac surgery (Group-D). The group-A patients had sick sinus syndrome or atrioventricular conduction disturbances more frequently than the other patients (2/3 vs. 3/18, *p* = 0.06). The group-C patients were older than the other group patients (78.2 ± 3.9 vs. 67.6 ± 13.1 years, *p* = 0.13). In group-B, a complete roof line block was achieved in all patients during the previous procedures.

**Table 2:** Clinical and procedural characteristics of the patients in the 4 groups

	Group-A	Group-B	Group-C	Group-D	*p* value
Patient, n	3	12	4	2	
Age, years	62.6 ± 20.4	69.2 ± 12.2	78.2 ± 3.9	66.0 ± 12.7	0.419
Male gender, n (%)	1 (33.3%)	8 (66.7%)	3 (75.0%)	0 (0%)	0.228
Concomitant AF, n (%)	3 (100%)	12 (100%)	4 (100%)	1 (50.0%)	0.019
Left atrial diameter, mm	36.7 ± 5.9	42.3 ± 6.8	45.8 ± 3.3	46.7 ± 3.9	0.217
Left ventricular ejection fraction, %	59.9 ± 9.4	64.9 ± 7.2	51.6 ± 21.5	44.0 ± 29.2	0.125
Structural heart disease, n (%)	1 (33.3%)	1 (8.3%)	1 (25.0%)	2 (100%)	0.043
SSS/AV conduction disturbance, n (%)	2 (66.7%)	2 (16.7%)	0 (0%)	1 (50%)	0.146
LA roof-dependent ATs, n	3	13	4	2	
The number of ablation procedure					0.004
1st procedure, n (%)	3 (100%)	0 (0%)	0 (0%)	2 (100%)	
2nd procedure, n (%)	0 (0%)	5 (38.5%)	3 (75.0%)	0 (0%)	
3rd procedure, n (%)	0 (0%)	7 (53.8%)	1 (25.0%)	0 (0%)	
4th procedure, n (%)	0 (0%)	1 (7.7%)	0 (0%)	0 (0%)	
Multiple ATs, n (%)	3 (100%)	6 (46.2%)	2 (50.0%)	2 (100%)	0.212
Concomitant peri-mitral ATs, n (%)	3 (100%)	2 (15.4%)	2 (50.0%)	2 (100%)	0.012
Length of LA roof between upper PVs, cm	23.0 ± 1.2	25.3 ± 3.8	29.6 ± 2.5	26.7 ± 0.1	0.084
Tachycardia cycle length, ms	210 ± 25	249 ± 47	230 ± 24	289 ± 28	0.205
Mean conduction velocity (CV), cm/s	86.1 ± 23.9	67.0 ± 13.4	79.7 ± 8.7	70	0.19
CV at slow conduction area, cm/s	7.8 (7.0–32.5)	7.7 (4.6–31.7)	13.2 (6.5–30.9)	17.4 (7.5–27.3)	0.992
Clockwise roof-dependent AT, n (%)	2 (66.7%)	7 (53.8%)	3 (75.0%)	1 (50.0%)	0.872
Roof-dependent AT around RPVs, n (%)	2 (66.7%)	13 (100%)	2 (50.0%)	2 (100%)	0.003
Roof-dependent AT around LPVs, n (%)	1 (33.3%)	0 (0%)	4 (100%)	0 (0%)	
LVA at LA roof, n (%)	2 (66.7%)	13 (100%)	4 (100%)	2 (100%)	0.085
LVA at LA roof, cm^2^	2.5 (0–4.1)	3.8 (3.0–4.4)	2.7 (2.0–4.1)	5.0 (4.5–5.6)	0.158
LVA at LA anterior/septal wall, n (%)	2 (66.7%)	7 (53.8%)	4 (100%)	1 (50.0%)	0.392
LVA at LA anterior/septal wall, cm^2^	1.4 (0–8.9)	2.8 (0–5.1)	4.6 (3.3–6.3)	2.9 (0–5.8)	0.846
LVA at LA posterior wall, n (%)	0 (0%)	7 (53.8%)	2 (50.0%)	2 (100%)	0.166
LVA at LA posterior wall, cm^2^	0 (0%)	2.7 (0–5.8)	0.4 (0–1.8)	1.2 (1.0–1.5)	0.249

Values are reported as the mean ± standard deviation, median [25th, 75th percentiles], or number of patients (%) unless otherwise noted

*AF* atrial fibrillation, *AT* atrial tachycardia, *AV* atrioventricular, *CV* conduction velocity, *LA* left atrium, *L(R)PVs* left (right) PVs, *LVA* low voltage area, *n* number, *PV* pulmonary vein, *SSS* sick sinus syndrome

### Mapping of the ATs

**Fig. 1:**
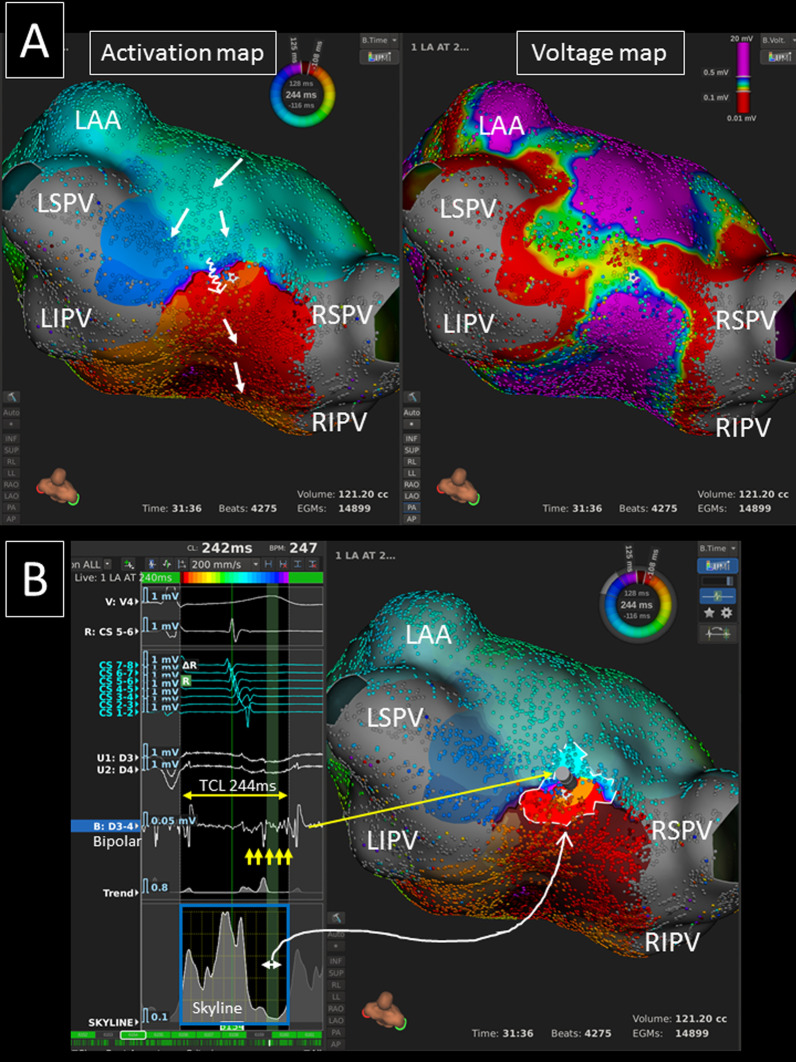
**A** An ultra-high resolution activation map revealed a clockwise roof-dependent AT with slow conduction on the LA roof (TCL = 244 ms) (left panel). The white arrows show the propagation of the activation. A voltage map shows an LVA on the LA roof (right panel). **B** The valley (green curtain, white arrow) on the global activation histogram (*skyline*, blue square) corresponds to the isthmus of the AT (highlighted area). Please note that low-voltage fractionated signals (yellow arrows) during the time period of the valley of the *skyline* were identified in the critical isthmus. *LAA* left atrial appendage, *LI(S)PV* left inferior (superior) pulmonary vein, *RI(S)PV* right inferior (superior) pulmonary vein, *TCL* tachycardia cycle length

**Fig. 2:**
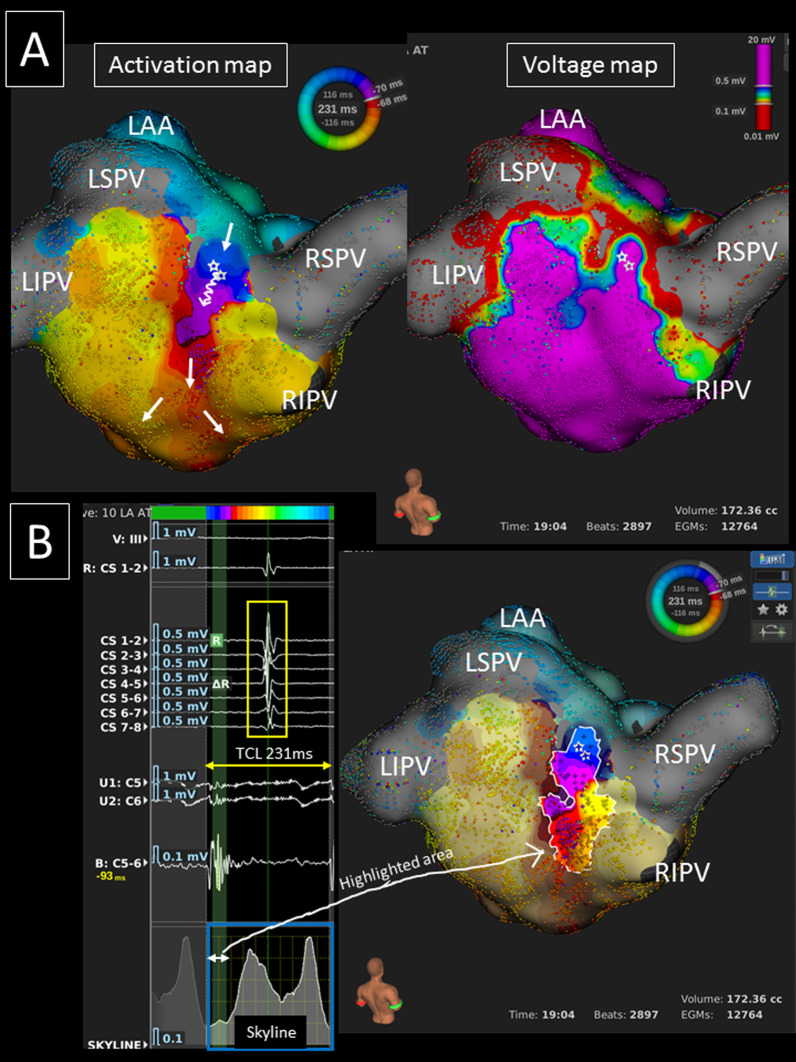
**A** An ultra-high resolution activation map revealed a clockwise roof-dependent AT with slow conduction on the LA posterior roof (TCL = 231 ms) (left panel). The white arrows show the propagation of the activation. A voltage map shows scar on the LA posterior roof (right panel). **B** The valley (green curtain, white arrow) on the global activation histogram (*skyline*, blue square) corresponds to the isthmus of the AT (highlighted area). Please note the straight CS activation pattern (yellow square). *LAA* left atrial appendage, *LI(S)PV* left inferior (superior) pulmonary vein, *RI(S)PV* right inferior (superior) pulmonary vein, *TCL* tachycardia cycle length

**Fig. 3:**
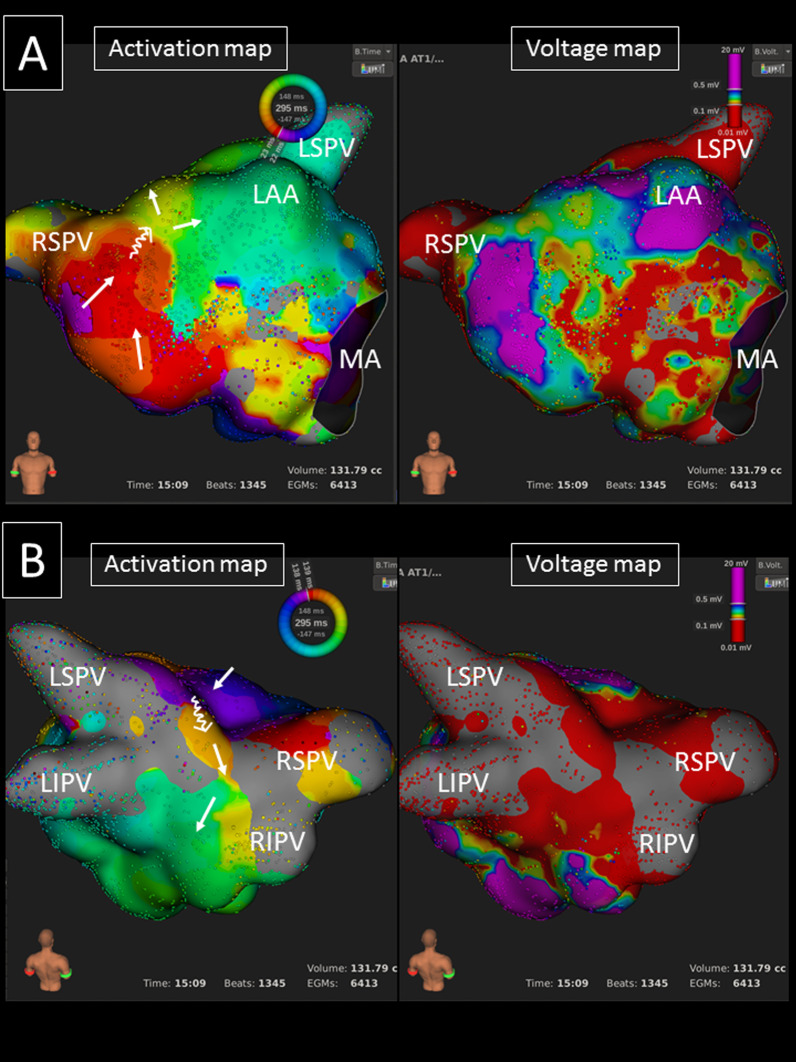
An ultra-high resolution activation map revealed a clockwise roof-dependent AT with slow conduction on both the LA anterior wall (**A**) and roof (**B**) (TCL = 295 ms) (left panels). The white arrows show the propagation of the activation. A voltage map reveals extensive scar and an LVA on the LA anterior (**A**) and posterior walls (**B**) (right panels). *LAA* left atrial appendage, *LI(S)PV* left inferior (superior) pulmonary vein, *MA* mitral annulus, *RI(S)PV* right inferior (superior) pulmonary vein

**Fig. 4:**
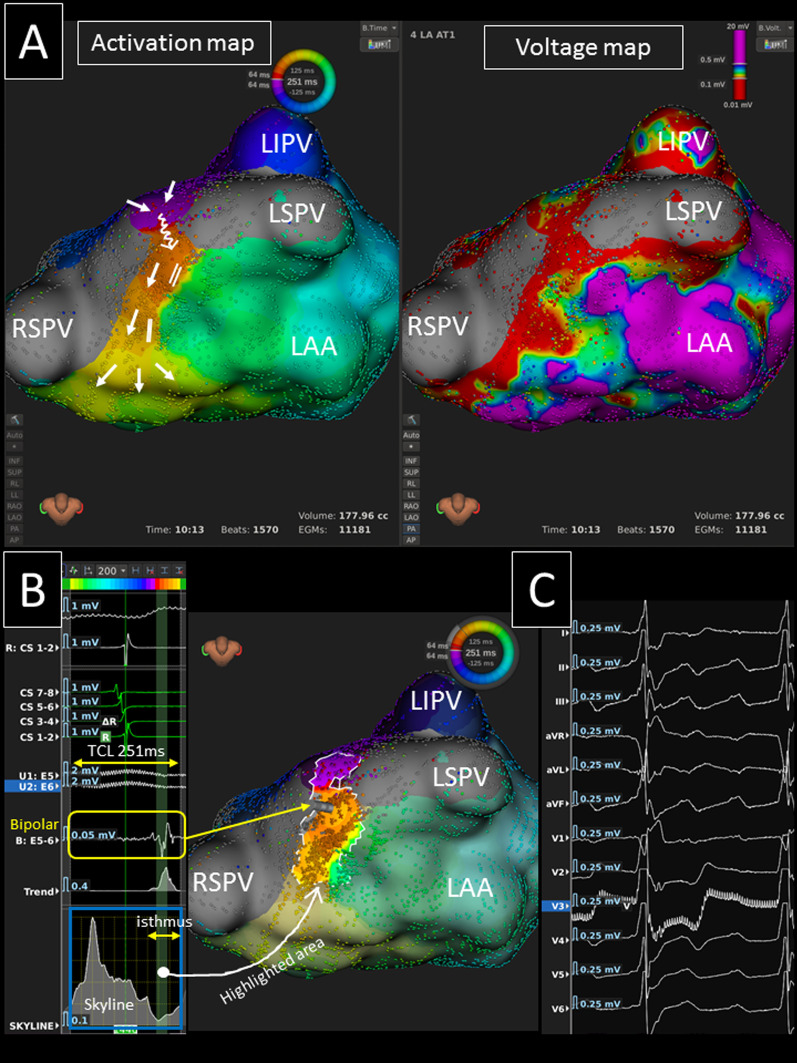
**A** An ultra-high resolution activation map revealed a counter-clockwise roof-dependent AT with a conduction path and slow conduction on the LA roof (TCL = 251 ms) (left panel). The white arrows show the propagation of the activation. A voltage map reveals scar on the LA roof (right panel). **B** The valley (green curtain, white arrow) on the global activation histogram (*skyline*, blue square) corresponds to the isthmus of the AT (highlighted area). Please note that fractionated signals (yellow square) during the time period of the valley of the *skyline* were identified in the critical isthmus. **C** During the AT, continuous electrical activity was observed on the 12-lead ECG. *LAA* left atrial appendage, *LI(S)PV* left inferior (superior) pulmonary vein, *RSPV* right superior pulmonary vein, *TCL* tachycardia cycle length

**Fig. 5:**
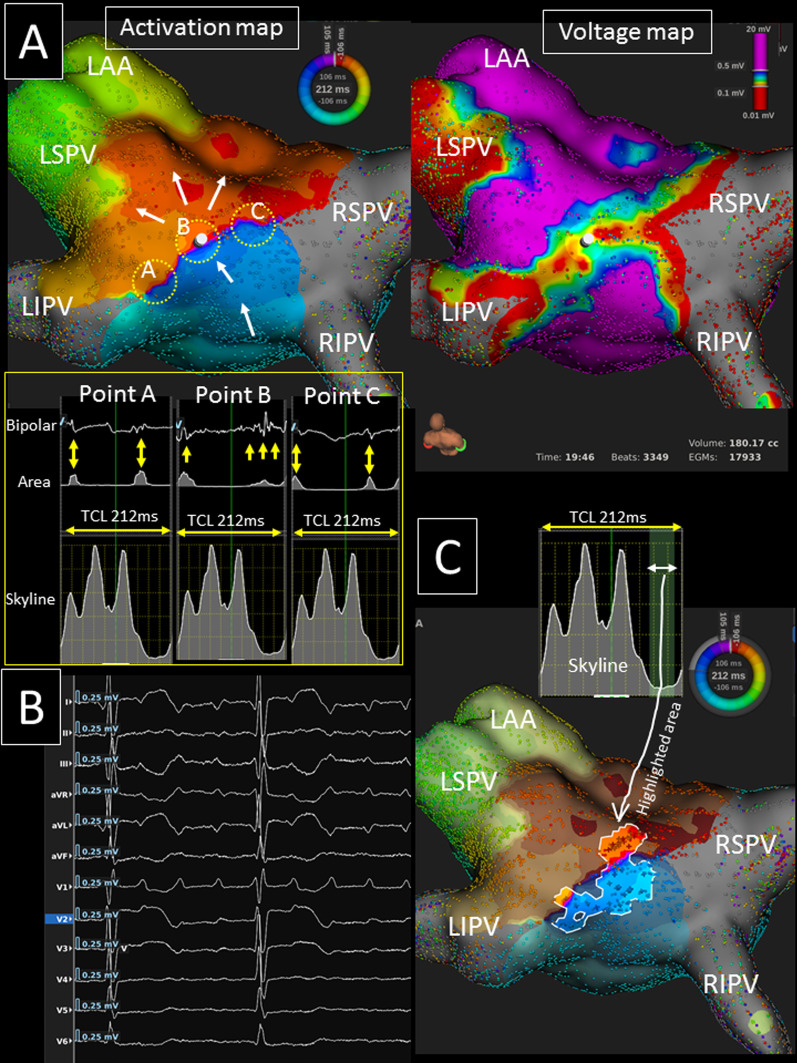
**A** An ultra-high resolution activation map revealed a counter-clockwise roof-dependent AT (TCL = 212 ms) (left panel). The white arrows show the propagation of the activation. A voltage map reveals an LVA on the LA posterior roof (right panel). Please note that double potentials are observed at point-A and point-C, but a low-amplitude fractionated signal is identified during the time period of the valley of the *skyline* at point-B (yellow square). **B** A 12-lead ECG during the AT exhibits a synchronous isoelectric interval. **C** The valley (green curtain, white arrow) on the global activation histogram (*skyline*) corresponds to the isthmus of the AT (highlighted area). *LAA* left atrial appendage, *LI(S)PV* left inferior (superior) pulmonary vein, *RI(S)PV* right inferior (superior) pulmonary vein, *TCL* tachycardia cycle length

The mean AT cycle length was 244 ± 43 ms, and activation maps of 10,410 (7643–15,732) points were acquired during the AT over a 15.2 (10.0–20.8) minute mapping time. Of the 22 roof-dependent ATs, the coronary sinus (CS) activation sequence was proximal-to-distal, distal-to-proximal, and a straight pattern in 13, 3, and 6 Ats, respectively. The activation map revealed 13 (59.1%) clockwise (cranio-caudal direction on LA posterior wall) (Figs. 1, 2, 3) and 9 (40.9%) counter-clockwise (cranio-caudal direction on LA anterior wall) (Figs. 4, 5) roof-dependent ATs. The mean length of LA roof line connecting the upper PVs was 25.9 ± 3.7 mm.

**Fig. 6:**
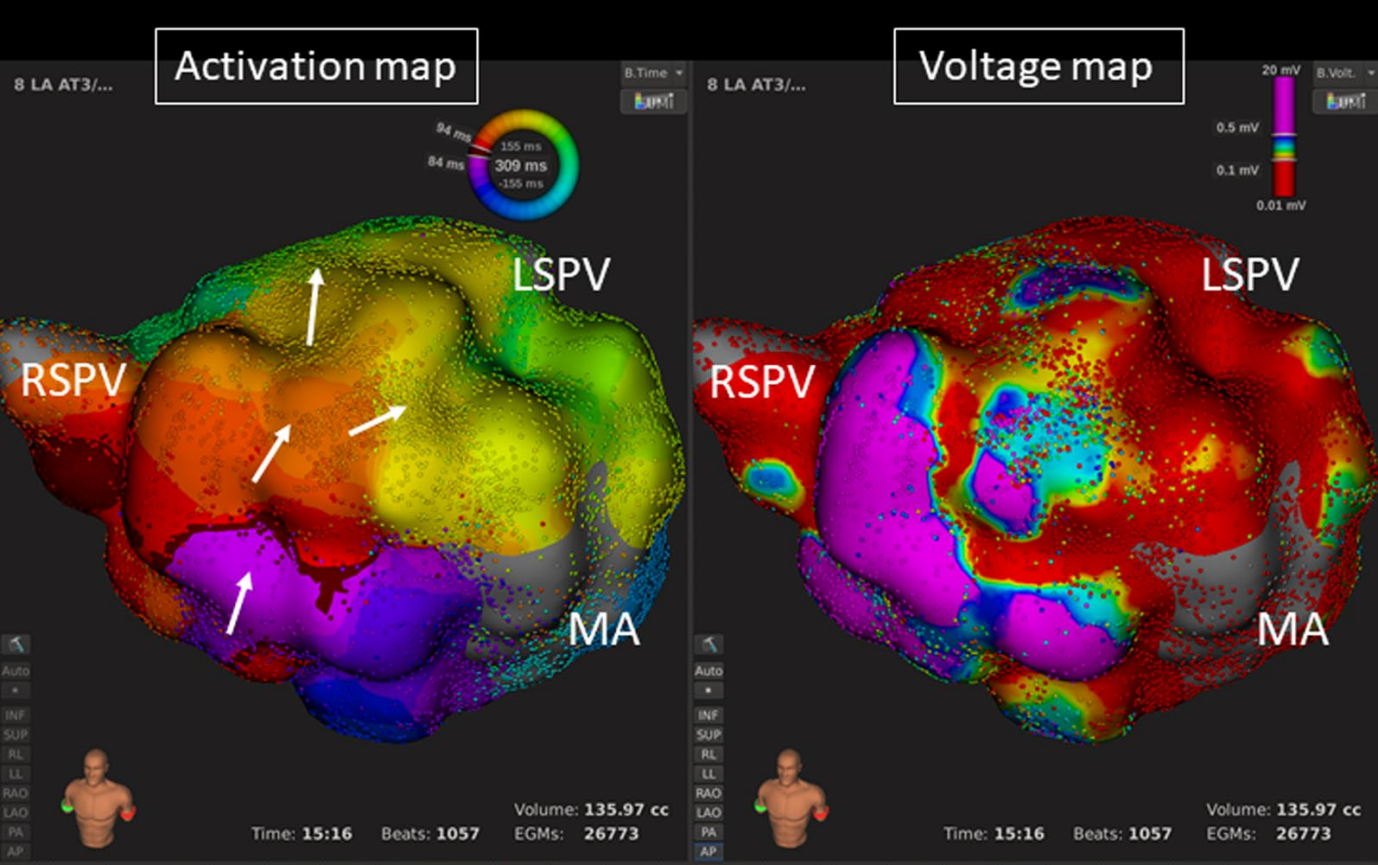
An ultra-high resolution activation map revealed a clockwise roof-dependent AT (TCL = 309 ms) (left panel) in a patient with a history of cardiac surgery. The white arrows show the propagation of the activation. A voltage map reveals an extensive LVA on the LA anterior wall (right panel). *LAA* left atrial appendage, *LI(S)PV* left inferior (superior) pulmonary vein, *MA* mitral annulus, *RI(S)PV* right inferior (superior) pulmonary vein

In the maps of the ATs, LVAs (< 0.5 mV) were identified on the LA roof, anterior/septal wall (Figs. 3A, 6), and posterior wall in 21 (95.4%), 14 (63.6%), and 11 (50.0%) ATs, and the area was a median of 3.7 (2.6–4.5), 3.3 (0–6.0), and 0.4 (0–3.9) cm^2^, respectively. All the group-A patients had LVAs on the LA roof and/or anterior/septal wall, but not on the posterior wall. All the group-C patients had LVAs on both the LA roof and anterior/septal wall. Group-B patents tended to have larger LVAs on the LA roof as compared to the group-A and group-C patients (Table 2).

The mean conduction velocity of the entire circuit was 72.5 ± 15.3 cm/s, and the mean conduction velocity in the slowest conduction area was 8.2 (5.8–30.0) cm/s. Slow conduction areas were identified on the LA roof, LA anterior/septal wall, and LA posterior wall in 18, 6 (2 in group-A and group-C, 1 in group-B and group-D), and 2 ATs, respectively. Thus, it was significantly more frequently identified in areas other than the LA roof in non-group-B than group-B patients (5/9 vs. 1/12, *p* = 0.018). All the global activation histograms (*Skyline* module in the *Lumipoint* software) in the patients with a slow conduction area had peaks and valleys. The duration of the valleys on the histograms highlighted the area of the critical isthmus in all ATs (Figs. 1, 2, 4, 5).

### P wave morphology

The P wave morphology during roof-dependent ATs could be evaluated in 19 ATs. The maximal P wave amplitude and duration on the 12-lead electrocardiogram (ECG) were 0.15 (0.10–0.19) mV and 89 (80–112) ms, respectively (Fig. 5B). In 10/19 (52.6%) ATs, a 12-lead synchronous isoelectric interval was observed, but not in the remaining 9/19 (47.4%) ATs (Fig. 4C). The isoelectric interval was significantly more frequently observed in group-B than group-A and group-C (8/11 vs. 1/7, *p* = 0.016).

### Catheter ablation

A mean of 1.9 ± 0.8 ATs were identified per procedure, and multiple ATs were observed in 13 (59.1%) procedures. An LA roof-dependent AT was the first and second AT during the procedure in 19 (86.4%) and 3 (13.6%) patients, respectively. An LA roof line ablation was performed in all. Sustained roof-dependent ATs were terminated by a single application and linear ablation in 6 (27.3%) and 9 (40.9%) ATs. In the remaining 7 (31.8%) ATs, AT converted to another AT during the roof line ablation. The roof line ablation resulted in complete conduction block in 18 (71.8%) procedures, however, it did not in 4 (18.2%) procedures. All 4 procedures were in group-B and they had extensive scar on the LA roof and high posterior wall (Fig. 3B). The roof line ablation inside the scar terminated the ATs, but linear block was not achieved despite multiple applications inside scar. Finally, linear ablation at the middle level of the LA posterior wall (lower than the scar) successfully resulted in complete linear block of that connecting the left and right PVs.

In group-B, the conduction gaps on the roof line were located close to the left PVs, mid-roof, and close to the right PVs, in 2 (15.4%), 7 (53.8%), and 4 (30.8%) procedures, respectively. A total of 20 concomitant ATs including a peri-mitral AT, LA anterior wall AT, ridge-related AT, localized AT, and focal AT in 9, 1, 2, 6, and 2 procedures, respectively, were identified by the ultra-high resolution mapping system. All concomitant ATs were successfully eliminated by creating linear lesions or targeting the critical isthmus. All the group-A and group-D roof-dependent ATs had concomitant ATs, and peri-mitral ATs were identified more frequently in non-group-B than group-B (7/9 vs. 2/13, *p* = 0.003) (Table 2). Since all group-B patients underwent mitral isthmus ablation previously, eventually mitral isthmus linear block was created in 19 out of 21 (90.5%) patients. No procedure-related complications were observed.

### Clinical outcomes

During 14.0 (6.5–28.5) months of follow-up period, 17/21 (81.0%) patients were free from any ATs after a single procedure. Among them, 4 patients took the same dose of antiarrhythmic drugs as before the procedure to control AF. Recurrent AF and AT were observed in one patient each, which were controlled with antiarrhythmic drugs. The remaining 2 patients exhibited recurrent stable ATs and underwent repeat procedures 2 months after the procedure. One was a peri-mitral AT in a patient without a history of a previous mitral isthmus ablation, and the other was a recurrent roof-dependent AT (including in this study) in a patient with active cardiac sarcoidosis. Both ATs were successfully eliminated, and they became free from any atrial arrhythmias after the repeat procedure. Thus, 19/21 (90.5%) patients were free from any ATs after the last procedure.

At the last follow-up, the LA diameter decreased (42.6 ± 6.4 vs. 41.4 ± 7.2 mm, *p* = 0.43), left ventricular ejection fraction increased (58.8 ± 14.4 vs. 64.2 ± 12.3%, *p* = 0.06), and serum brain natriuretic peptide level significantly decreased (89.1 [48.0–157.0] vs. 53.6 [31.0–78.8] pg/ml, *p* = 0.03) as compared to that prior to the procedure.

## Discussion

We found that (1) ultra-high resolution mapping with the *Lumipoint* algorithm successfully identified the critical isthmus of the ATs and lead to a high success rate, (2) almost all patients had AF, and peri-mitral ATs often coexisted with roof-dependent ATs, (3) slow conduction areas were located not only on the LA roof but also on the anterior/septal and/or posterior wall, (4) the majority had a history of a previous LA roof line ablation with the achievement of conduction block, (5) ablation on the mid-posterior wall was required to achieve complete linear block in approximately one fifth of ATs, (6) sinus node dysfunction or atrioventricular conduction disturbances often coexisted in intervention naïve patients, (7) approximately half of the ATs had isoelectric intervals on 12-lead ECG, and (8) conduction gaps contributing to the roof-dependent AT were predominantly located on the mid-roof.

### The mechanisms of roof-dependent ATs

Previous LA linear ablation increased the risk of recurrent LA macroreentrant ATs [1], and approximately half of the ATs were post roof-line ablation (group-B patients) in this study. As compared to group-B, group-A patients had anterior/septal wall LVAs more frequently and a high prevalence of conduction diseases, suggesting the presence of underlying atrial cardiomyopathy. As compared to group-B, group-C patients were older and had anterior/septal wall LVAs more frequently, suggesting a relatively advanced atrial cardiomyopathy. In addition, slow conduction areas were identified not only on the LA roof but also on the anterior/septal and posterior walls. It was significantly more frequently identified at other sites than the LA roof in non-group-B patients than group-B patients. All these data suggested that (1) a roof line ablation increased the risk of a roof-dependent AT due to a conduction gap and (2) slow conduction due to atrial disease on the LA anterior/septal and posterior wall contributed to the maintenance of roof-dependent ATs even without a history of linear ablation. We assume that AF was the trigger and the slow conduction was the substrate of roof-dependent ATs given that most of the patients had concomitant AF.

The other interesting findings were that 7 patients exhibited concomitant peri-mitral ATs among 9 patients without a history of a mitral isthmus linear ablation (non-group-B patients). As a result, mitral isthmus linear block was created in 19 out of 21 patients in this study. Since the presence of LVAs on the LA anterior/septal wall contributed to the occurrence of macroreentrant LA ATs, both LA roof and mitral isthmus linear ablation might be required in those populations. We think that the low rate of recurrent ATs in this study was due to the high durability of the linear conduction block and elimination of all concomitant ATs.

Contact force sensing-catheters were not available for use with the Rhythmia mapping system during this study period. However, we believe that the precise identification of the critical isthmus was the most important point rather than the presence of a contact force sensing-catheter for the treatment of ATs. The high AF/AT freedom after eliminating the Ats observed in this study supported our hypothesis.

### LA roof line ablation

In our study, all the sustained roof-dependent ATs were eliminated by a roof line ablation, however, complete linear block could not be created on the LA roof area in 18% of the procedures. All these patients had scar on the LA roof and high posterior wall, and eventually complete linear block was achieved on the mid-posterior wall. We speculated that this was due to an epicardial connection bridging the LA roof scar area. Indeed, the superior LA has the most substantial thickness of the LA and is between 3 and 6.5 mm thick in normal human hearts [15]. All the group-B patients had a complete roof line block during the previous session, indicating the difficulty of creating a durable lesion.

The location of conduction gaps on the LA roof line was investigated without the use of a 3-D mapping system previously, and the line predominantly recovered close to the right superior PV and less frequently on the mid-roof or close to the left PV [16]. On the contrary, our data showed that the majority of the conduction gaps related to recurrent roof-dependent ATs were located on the mid-roof. The difference may be explained by the different study populations (only clinical roof-dependent ATs were investigated in our study), and the progression of the ablation technology in creating linear lesions.

### P wave morphology and CS activation sequence

Generally, a wide isthmus reentry shows continuous electrical activity on the surface ECG. However, a narrow isthmus reentry with significant conduction delay demonstrates discrete P waves separated by a 12-lead synchronous isoelectric interval [17]. Our data that approximately half of the ATs had a 12-lead synchronous isoelectric interval indicated that the roof-dependent ATs could not be characterized by the P-wave morphology.

CS activation is the initially obtained electrical information in the mapping of ATs and facilitates reaching the diagnosis of the ATs. A previous study showed that ATs with the chevron/reverse chevron activation were mostly roof-dependent [18]. On the contrary, approximately 70% of roof-dependent ATs had a proximal-to-distal or distal-to-proximal CS activation in our study. The discrepancy might be explained by the different study populations (only clinical roof-dependent ATs were included in our study). Thus, the roof-dependent ATs could not be characterized by the CS activation sequence.

### Study limitations

This was a small single center study. The maps were created during tachycardias in most of the cases. Direction of the traveling wavefront may influence the voltage and therefore the mapping of slow conduction corridors. The conventional one direction mapping with the Orion catheter (not omnipolar technology) may have an impact on the study results.

## Conclusions

The ultra-high resolution mapping system is useful to identify the critical isthmus of the tachycardia and to eliminate LA roof-dependent ATs. The lumipoint module shows an area of potential interest in the ATs which is important aid for the physician, however the last word should be reserved to the electrophysiology. The arrhythmia mechanisms are distinct in the individual patients, and elimination of all concomitant ATs is required for a better clinical outcome.

## Data Availability

The data that support the findings of this study are available from the corresponding author upon reasonable request.

## Abbreviations

AT: Atrial tachycardia
CS: Coronary sinus
LA: Left atrium
PV: Pulmonary vein
